# Tracking a Decentralized Linear Trajectory in an Intermittent Observation Environment

**DOI:** 10.3390/s20072127

**Published:** 2020-04-09

**Authors:** Wasi Ullah, Irshad Hussain, Iram Shehzadi, Zahid Rahman, Peerapong Uthansakul

**Affiliations:** 1 Faculty of Electrical & Computer Engineering, University of Engineering and Technology, Peshawar 25000, Pakistan; engr.wasiullah@uetpeshawar.edu.pk (W.U.); ee.irshad@gmail.com (I.H.); iramrvc@gmail.com (I.S.); 2 Department of Electrical Engineering, University of Science & Technology, Bannu 28100, Pakistan; zahidrahmanustb@gmail.com; 3 School of Telecommunication Engineering, Suranaree University of Technology, Nakhon Ratchasima 30000, Thailand

**Keywords:** decentralized cyber-physical system, linear trajectory, fault detection and isolation (FDI) algorithms, Kalman filter, tracking, sensor network

## Abstract

Faults and failures are familiar case studies in centralized and decentralized tracking systems. The processing of sensor data becomes more severe in the presence of faults/failures and/or noise. Effective schemes have been presented for decentralized systems, in the presence of faults only. In some practical scenarios of systems, there are certain interruptions in addition to these faults. These interruptions may occur in the form of noise. However it is expected that the decision about the sensor data is difficult in the presence of noise. This is because the noise adversely affects the communication amongst sensors and the processing unit. More complexity is expected when there are faults and noise simultaneously. To deal with this problem, in addition to existing fault detection and isolation schemes, the Kalman filter is employed. Here, a generic discussion is provided, which is equally applicable to other situations. This work addresses various faults in the presence of noise for decentralized tracking systems. Local single faults and multiple faults in the presence of noise are the core issues addressed in this paper. The proposed work is comprised of a general scenario for a decentralized tracking system followed by a case study of a target tracking scenario with and without noise. The presented schemes are also tested for different types of faults. The proposed work presents effective tracking in the presence of noise and faults. The results obtained demonstrate the acceptable performance of the scheme of this work.

## 1. Introduction and Background

Decentralized systems in the field of tracking are an emerging area of research. The mentioned systems are highly dependent on sensor information. Sensors measure physical quantities and transduce them into signals, which are used for further processing and control purposes [1]. As a matter of fact, sensors are electronic devices that are prone to faults and failures. These faults and failures mislead the system. In addition to that, noise is also an interrupting factor. To avoid these faults and failures, state estimation is an acceptable tool [2]. The distinctive property of state estimation is that it is efficient for noise of all frequencies. In the scenarios where ordinary filtering fails, state estimation is employed. Amongst state estimation techniques, the Kalman filter is one of the frequently used schemes [3]. Nonetheless, the extraction of accurate system information from noisy sensed measurements is the fundamental goal of the Kalman filter [4]. The sensor readings are used to compute the minimum mean squared error estimate of the system state. There are several estimation methods that are suitable for linear, nonlinear, and decentralized tracking systems. State estimation for Linear Time Invariant (LTI) systems used in mutual tracking is an attractive area of research due to its wide applications.

The predominant application of state estimation is a combined tracking system, which benefits from the distinct perspectives of data provided by a multi-sensor network. In order to provide more information, a large-scale multi-sensor network can be used. In order to estimate the position of the target by sensors, a multi-sensor network can be used, which demands adaptable signal processing. For this purpose, decentralized tracking based on state estimation has become an important research area to explore [5]. However, sensors are electronic devices and are susceptible to fault and breakdown/failures, which impacts the decentralization of the system. An important function of a typical sensor network is to detect and interpret actual system parameters, e.g., environmental monitoring, tanks, land mines, security, military, etc., in the presence of faulty sensor measurements [6]. In order to maintain the integrity of a decentralized system, the sensors must be secure, reliable, and available at a suitable location. In modern flight control systems, sensor failures may cause serious problems, which need to be accurately detected and addressed effectively and as soon as possible. To avoid system failure due to faulty sensors, the system must be able to handle sensor operation in faulty environments [7].

The literature reveals that Distributed Particle Filtering (DPF) is being used in mutual tracking [8]. In addition to that, the distributed fusion rule based on Bayes’ theorem [8] has been derived for tracking. Various schemes have been presented for Fault Detection and Isolation (FDI) in uninterrupted environments [9]. However, in noisy environments, existing approaches fail to track the mutual system. In this connection, algorithms in noisy environments use estimation schemes like the Kalman filter for estimation [9]. Moreover, recursive optimal Bayesian estimation is a statistical approach to track the mutual system. Generally, a recursive Bayesian estimation algorithm sequentially updates current state estimates based on its previous estimate and the incoming data from the sensors, it is also called a Minimum Mean-Squared Error (MMSE) estimator. The Kalman filter is also considered as one of the most popular recursive Bayesian estimation algorithms and has been successfully used in various applications, including all forms of nuclear power plant instrumentation, navigation (aerospace, land, and marine), manufacturing, demographic modeling, the detection of underground radioactivity, and neural network training [5,10].

To handle various types of faults associated with mutual tracking of a linear trajectory, some Fault Detection and Isolation (FDI) algorithms have been presented [7]. These algorithm work efficiently in the absence of noise, but this is hard to achieve in an ideal environment practically. Therefore, a relatively practical and realistic approach is adopted that will effectively track the linear trajectory in the presence of noise and faults simultaneously.

This work presents strategies for practical scenarios of target tracking when the system is subjected to simultaneous faults/failure in a noisy environment, in contrast with the existing work, where one fault or failure has been handled in a non-noisy environment. To put it simply, Kalman filtering has been suggested to be suitable for the above-mentioned case of study, and algorithms have been proposed for coping with intermittent output data caused by sensor faults and noise. Employing these algorithms provides the successful completion of the mission in a specific formation F.

The paper commences with the Introduction followed by the generalized system model with and without interruption. In the next section, the Kalman filter is presented, and then, the results of the proposed scheme are presented. Finally, the paper is summarized, followed by references.

## 2. Decentralized System Model

A generalized mutual tracking scenario is represented in the state space model, where *x* and *y* are components of the trajectory taken to be the states of the system. As in tracking, both the *x* and *y* components of the linear trajectory are measured using numerous sensors. The target is following a linear trajectory in Cartesian coordinates, and both components change as it follows its path. Follower UAVs track that movement and hence update its position accordingly. All the system dynamics are defined as per the basic rules of the control system. The standard state space representation of the tracking scenario is as follows.

(1) $$x_{k+1|k}=Ax_{k|k}+Bu_{k}$$

The system dynamics, state vector, and input vectors are defined. The state equation presents the position of the target and its movement in a linear trajectory.

(2) $$x\left(i,k+1\right)=\left(1-\gamma \right)x\left(i,k\right)+b_{1i}dt$$

where:

i=1,2 and γ is a constant parameter and is defined as 0<γ<1 for the stability constraints of the system. $b_{1i}$ is a square matrix based on the mutual dependance of system parameters.

The output equation is given as:

(3) $$y_{k+1|k}=Cx_{k+1|k}$$

$$\begin{array}{c} \begin{array}{c} \left[\begin{array}{c} y(1,k+1)\\ y(2,k+1) \end{array}\right]=\left[\begin{array}{cc} 1 & 0\\ 0 & 1 \end{array}\right]\left[\begin{array}{c} x(1,k)\\ x(2,k) \end{array}\right] \end{array} \end{array}$$

where the **C** matrix is the output vector.

It is assumed that both *x* and *y* components of the trajectory are measured. These are the dimensions of all the system dynamics and vectors as shown: $A:R^{z}⟶R^{m\times m}$ is the system transition matrix; $B:R^{z}⟶R^{m\times m}$ is the input matrix; $C:R^{z}⟶R^{q\times m}$ is the output matrix; $x_{k}:R^{z}⟶R^{m\times 1}$ represents the state of the system; $u_{k}:R^{z}⟶R^{q\times 1}$ represents the deterministic input; $y_{k}:R^{z}⟶R^{q\times 1}$ is the output of the system.

## 3. Decentralized System Model with Interruption

In the field of tracking, sensors are of great importance as they are the key components to compute the information of the trajectory. The sensors are vulnerable electronic devices that may fail any time or their readings may not be accurate. In addition to that, communication may be affected by unwanted environmental signals. To observe the effects of all these unforeseen conditions, intermittency is introduced. The state space model will be:

(4) $$x_{k+1|k}=Ax_{k|k}+Bu_{k}+w_{k}$$

In the process equation, $w_{k}:R^{s}⟶R^{n\times 1}$ is assumed to be the interruption in the process. The interruption in the decentralized system will be:

$$\begin{array}{c} \begin{array}{c} \left[\begin{array}{c} x(1,k+1)\\ x(2,k+1) \end{array}\right]=\left[\begin{array}{cc} 1-\gamma & 0\\ 0 & 1-\gamma \end{array}\right]\left[\begin{array}{c} x(1,k)\\ x(2,k) \end{array}\right]+\left[\begin{array}{cc} b_{11} & 0\\ 0 & b_{12} \end{array}\right]\left[\begin{array}{c} dt\\ dt \end{array}\right]+\left[\begin{array}{c} w(1,k)\\ w(2,k) \end{array}\right] \end{array} \end{array}$$

where w(1,k) is the process error in the *x* component of the trajectory. w(2,k) is the process error in the *y* component of the trajectory.

The measurement equation is given by:

(5) $$y_{k+1|k}=Cx_{k+1|k}+v_{k}$$

In the measurement equation, $v_{k}:R^{s}⟶R^{q\times 1}$ is assumed to be the intermittency in the measurement. For the decentralized systems, it becomes:

$$\begin{array}{c} \begin{array}{ccc} \left[\begin{array}{c} y(1,k+1)\\ y(2,k+1) \end{array}\right]=\left[\begin{array}{cc} 1 & 0\\ 0 & 1 \end{array}\right]\left[\begin{array}{c} x(1,k)\\ x(2,k) \end{array}\right] & + & \left[\begin{array}{c} v(1,k)\\ v(2,k) \end{array}\right] \end{array} \end{array}$$

where: v(1,k) is the measurement uncertainty in the *x* component of the trajectory. v(2,k) is the measurement uncertainty in the *y* component of the trajectory.

The measurement uncertainty can be described as the error caused by the inaccuracy of our instruments. It may be an unwanted signal added to the measurement by an unknown exogenous and/or endogenous source.

The vectors w and v are assumed to have the following properties:

Interruptions are assumed to be random signals with flat spectral power density.

$w\left(k\right):R^{s}⟶R^{m\times 1}$ and $v\left(k\right):R^{s}⟶R^{q\times 1}$ are the process and measurement interruption vectors, respectively.

These are also assumed to be uncorrelated, white Gaussian, zero mean, and bounded covariance matrices as $w\left(k\right)∼N\left(0,Q_{k}\right)$ and $v\left(k\right)∼N\left(0,R_{k}\right)$, where $Q_{k}$ and $R_{k}$ are covariance matrices. Interruptions are uncorrelated with $u_{k}$ and $x_{k|k}$, having a joint covariance matrix given by,

(6) $$E\left[\begin{array}{c} w(k)\\ v(k) \end{array}\right]\left[\begin{array}{cc} w^{T}\left(z\right) & v^{T}\left(z\right) \end{array}\right]=\left[\begin{array}{cc} Q & Z\\ Z^{T} & R \end{array}\right]\delta_{kz}$$

where $Q\in R^{m\times m}$, $S\in R^{m\times q}$, and $R\in R^{q\times q}$ and in which $w_{k}:R^{z}⟶R^{m\times 1}$ is the process uncertainty and $v_{k}:R^{s}⟶R^{q\times 1}$ is the measurement uncertainty. These uncertainties may mislead the system to an unavoidable situation, which may be fatal for the decentralization of the system. To deal with this and to maintain the integrity of the mentioned system, a state estimator based on standard Kalman filtering is proposed.

## 4. State Estimator Based on Kalman Filtering

State estimation is an attractive tool for dealing with intermittent data. Important data can be efficiently retrieved using estimation. Effective schemes like the Unscented Kalman Filter (UKF) and the Particle Filter (PF) are proposed in the literature, which are finding application in nonlinear scenarios. However, they possess the complexity of nonlinearity, and they may be employed for a nonlinear trajectory [11]. An acceptable scheme in the state estimation for linear trajectory movement is the Kalman filter. The Kalman filter is the optimal estimator that provides an efficient computational (recursive) tool to estimate the parameters involved in tracking, when it is perturbed by errors. The Kalman filter is theoretically attractive apart from all other possible filters, because it is one that minimizes the variance of the estimation error [10]. Mathematically, the Kalman filter estimates the states of a linear system. Initially gain, noise covariance, and prediction covariance are assumed. Using these initial values, the Kalman gain was calculated and then the estimated value predicted to update the state and covariances. The estimates produced by this method made the actual values equal to the original measurements [12]. The following two basics steps were involved in the proposed estimation scheme:

- Prediction step
- Update step

These steps involved in estimation have a similar nature to Kalman filtering. The application of the scheme needs some necessary assumptions. The proposed scheme can be applied on an interrupted decentralized system model of tracking, with these two assumptions.

- Assumption 1: The system model of the tracking scenario should be Linear Time Invariant (LTI).
- Assumption 2: Both the process interruption and the measurement interruption are assumed to be zero mean and have flat spectral density. It is random, having a uniform distribution and being Gaussian in nature.

With the above two assumptions, the scheme is presented mathematically. The state equation is shown as,

$$\begin{array}{c} \begin{array}{c} \left[\begin{array}{c} x(1,k+1)\\ x(2,k+1) \end{array}\right]=\left[\begin{array}{cc} 1-\gamma & 0\\ 0 & 1-\gamma \end{array}\right]\left[\begin{array}{c} x(1,k)\\ x(2,k) \end{array}\right]+\left[\begin{array}{cc} b_{11} & 0\\ 0 & b_{12} \end{array}\right]\left[\begin{array}{c} dt\\ dt \end{array}\right]+\left[\begin{array}{c} w(1,k)\\ w(2,k) \end{array}\right] \end{array} \end{array}$$

These equations show an LTI system that is affected by the interrupted environment, to deal with this, standard steps involved in estimation are employed.

### 4.1. Prediction Step Equations

In the prediction step, the current state and error covariance estimates are projected forward to update the step via Kalman gain. The Kalman filter is modeled for the target tracking scenario by utilizing a linear algebra approach using matrices,

(7) $$x_{k+1|k+1}=Ax_{k+1|k}+Bu_{k}$$

This equation shows the predicted state, where $x_{k|k}$ is a vector representing the current state and $x_{k+1|k}$ is a matrix representing the updated state, which contains the x- and y-components of the vector position and the variable *k* represents the time step. The recursive process of the Kalman filter includes the use of epoch *k* to update the new position of the target through the state of one time step *k* to the next k+1 [13]. Taking the covariance of the error in measurement and prediction:

(8) $$P_{k+1|k}=AP_{k|k}A^{T}+Q_{k}$$

For the mentioned system, it will be:

$$\begin{array}{c} \begin{array}{c} \left[\begin{array}{cc} P_{11} & P_{12}\\ P_{21} & P_{22} \end{array}\right]=\left\{{\left[\begin{array}{cc} 1-\gamma & 0\\ 0 & 1-\gamma \end{array}\right]}^{2}\left[\begin{array}{cc} P_{11} & P_{12}\\ P_{21} & P_{22} \end{array}\right]\right\}+\left[\begin{array}{cc} Q_{11}\left(1,k\right) & 0\\ 0 & Q_{22}\left(2,k\right) \end{array}\right] \end{array} \end{array}$$

### 4.2. Update Step Equations

In the update step, predicted state $x_{k|k}$ and covariance $P_{k|k}$ estimates were updated by projecting forward epoch *k* to step k+1, in order to minimize the noise and estimation error effectively and to obtain improve predicted estimates [14,15].

(9) $$K_{k}=CP_{k+1|k}{\left[CP_{k+1|k}C^{T}+R_{k}\right]}^{-1}$$

For this system, it will be modeled as,

(10) $$\begin{array}{c} \left[\begin{array}{cc} K_{11} & K_{12}\\ K_{21} & K_{22} \end{array}\right]=\left[\begin{array}{cc} P_{11} & P_{12}\\ P_{21} & P_{22} \end{array}\right]\times H \end{array}$$

where:

(11) $$\begin{array}{c} H={\left\{\left[\begin{array}{cc} P_{11} & P_{12}\\ P_{21} & P_{22} \end{array}\right]+\left[\begin{array}{cc} R_{11}\left(1,k\right) & 0\\ 0 & R_{22}\left(2,k\right) \end{array}\right]\right\}}^{-1} \end{array}$$

This equation calculates the Kalman Gain $\left(y_{k}-Cx_{k+1|k}\right)$, which is basically the difference between the actual and predicted measurements; gain is responsible for more effectively correcting the predicted state by minimizing the estimation error and the effect of the noise that comes in the measurements. As time goes on, the measurement is weighted less due to the effect of the gain, so the update state has more improved estimates compared to the prediction step. The Kalman gain is bounded by the R and P matrices, where R is a covariance matrix of the measurement noise and P is a covariance matrix of the process noise [13,15]. The update equation will be:

(12) $$x_{k+1|k+1}=x_{k+1|k}+K_{k}\left[y_{k+1}-{\hat{y}}_{k+1|k}\right]$$

for the target tracking scenario, it will be,

(13) $$\begin{array}{c} \left[\begin{array}{c} x(1,k+1)\\ x(2,k+1) \end{array}\right]=\left[\begin{array}{c} x(1,k+1)\\ x(2,k+1) \end{array}\right]+KS \end{array}$$

where:

(14) $$\begin{array}{c} K=\left[\begin{array}{cc} K_{11} & K_{12}\\ K_{21} & K_{22} \end{array}\right] \end{array}$$

(15) $$\begin{array}{c} S=\left\{\left[\begin{array}{c} y(1,k+1)\\ y(2,k+1) \end{array}\right]-\left[\begin{array}{c} \hat{y}\left(1,k+1\right)\\ \hat{y}\left(2,k+1\right) \end{array}\right]\right\} \end{array}$$

Predicted state covariance matrix $\left(P_{k|k}\right)$ is updated from epoch *k* to k+1 to get the update of covariance matrix $\left(P_{k+1|k}\right)$, which is based on Kalman gain. The Kalman filter uses previous predicted data to predict the actual position of the target by filtering out the error result from the noise. The update covariance matrix has less estimation error compared to the predicted one [13].

(16) $$P_{k+1|k+1}=\left(I-K_{k+1}\right)P_{k+1|k}$$

therefore, it becomes,

(17) $$\begin{array}{c} \left[\begin{array}{cc} P_{11} & P_{12}\\ P_{21} & P_{22} \end{array}\right]=\left\{\left[\begin{array}{cc} 1 & 0\\ 0 & 1 \end{array}\right]-\left[\begin{array}{cc} K_{11} & K_{12}\\ K_{21} & K_{22} \end{array}\right]\right\}\times \left[\begin{array}{cc} P_{11} & P_{12}\\ P_{21} & P_{22} \end{array}\right] \end{array}$$

The above steps (prediction and update) are repeated recursively over time. The loop is initiated, and with a specified time step, it is incremented. The end time is also provided, which specifies the number of times of execution of the scheme. At each cycle, the intermittent states are fed to the Kalman filter, which estimate the state and are stored in a location. The stored estimated state values are then plotted for analysis. At each instant, the state is predicted, which is then updated via Kalman gain in the update step. The proposed model was tested in different environments, and the results were observed as shown in the Results Section.

## 5. Simulation and Results

This research work proceeds from the noise-free scenario to the noisy environment in which sensor data are subjected to Gaussian noise. The noise was generated randomly with different covariance and levels. The effect of noise with no fault and with Line Of Communication (LOC) and Communication with Neighbor (CN) faults simultaneously is shown in the upcoming figures.

The Kalman filter implementation in addition to the existing target tracking algorithm was the major part of this research work. As is clear from the literature, the Kalman filter estimates the state of an LTI system in the presence of noise. Here, the Kalman filter was employed to eliminate random deviations from the position and estimated the tracks of the target. The proposed scheme was tested in the presence of noise and faults. The defined scheme was tested in the absence of noise and faults as follows.

### 5.1. Eight Follower (UAVs) Noise-Free Scenario

**Figure 1:**
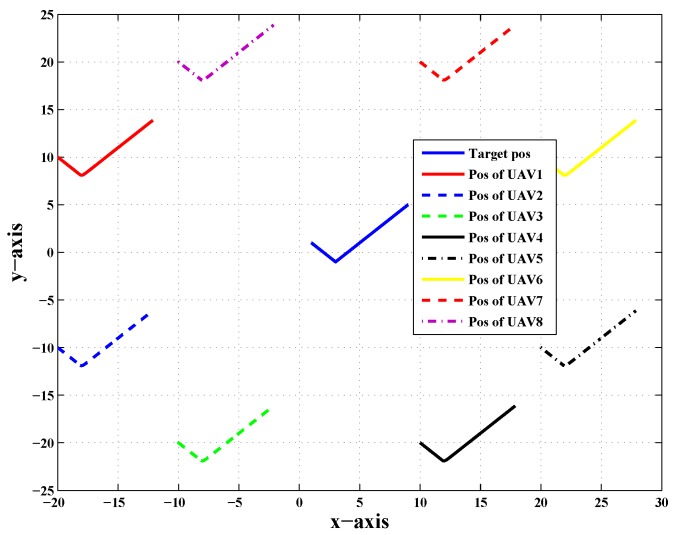
Top view of the target tracking scenario without noise and fault.

Target tracking is one of the most important research areas. A circular formation was taken into consideration to evaluate the proposed scheme. Figure 1 shows the top view of a target tracking scenario in which targets had their central trajectory tracked down by eight followers that encircled the target in the absence of noise and fault.

**Figure 2:**
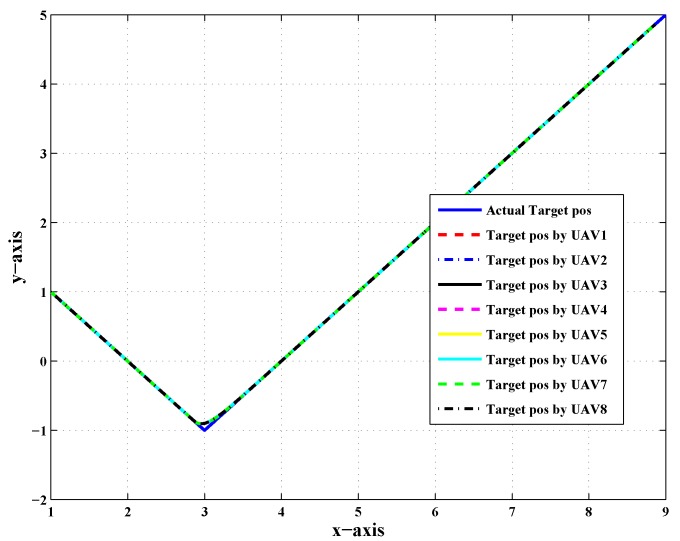
Combined trajectory of the actual target position and the position of eight UAVs

Figure 1 shows the top view of the target tracking scenario in which all UAVs were tracking the target efficiently in the absence of noise and fault, while Figure 2 shows the combined trajectories of the target and eight followers without fault and noise; the path followed by the target and its position estimated by each UAV are shown, which guaranteed that the track estimated by each UAV in the noise-free environment was acceptable.

The presented target tracking scenario was affected by faults and noise as discussed in the next section.

### 5.2. Eight Followers (UAVs) with LOC and CN Faults Simultaneously in the Presence of Noise

**Figure 3:**
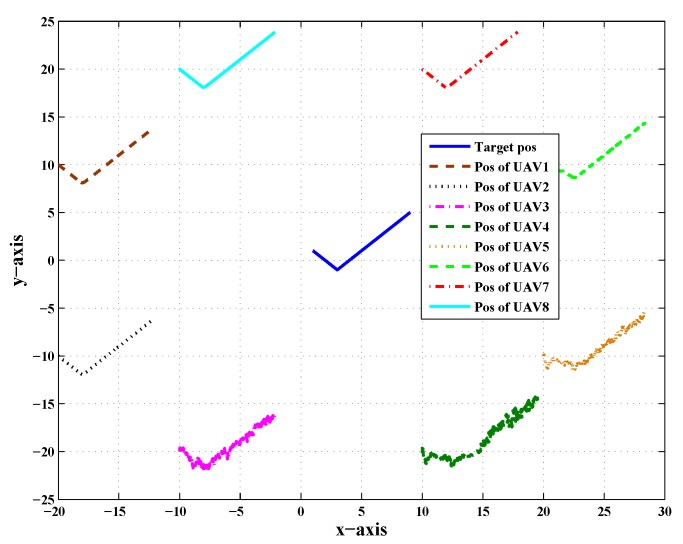
Top of view of the target and its followers in the presence of noise and Line Of Communication (LOC) and Communication with Neighbor (CN) faults simultaneously.

This section presents the effect of noise in the presence of Line Of Communication (LOC) and Communication with Neighbor (CN) faults simultaneously. Figure 3 shows the top view of target and its follower UAVs, which shows the presence of noise and the effect of both CN and LOC faults simultaneously. A CN fault was between UAV-3 and UAV-4, while an LOC fault was affecting UAV-5. The presence of both CN and LOC faults affected its neighbor trajectory due to which the UAVs went through an unusual path to follow the target, but somehow, they were able to track the target back due data fusion, FDI algorithms, and the consensus algorithm. The unusual path was due to the data fusion algorithm, which used the neighbor data for estimating the position. The LOC fault affected the calculation of the target position. It is shown in the figures that these faults affected the neighbor followers, which was because of the decentralized environment. However, when noise was added into the target position, the UAVs were completely unable to estimate the target position and its path, as shown.

**Figure 4:**
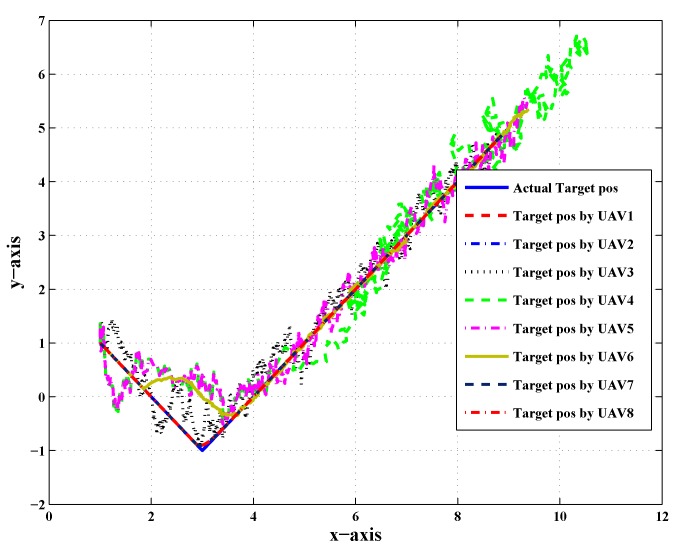
Target trajectory of LOC and CN faults in the presence of noise.

Figure 4 shows the combined trajectory of the target and the follower UAVs in the presence of noise and both CN and LOC faults; due to the effect of noise and both faults, the UAVs followed an unusual path to track the path of the target.

### 5.3. Kalman Filter Implementation

**Figure 5:**
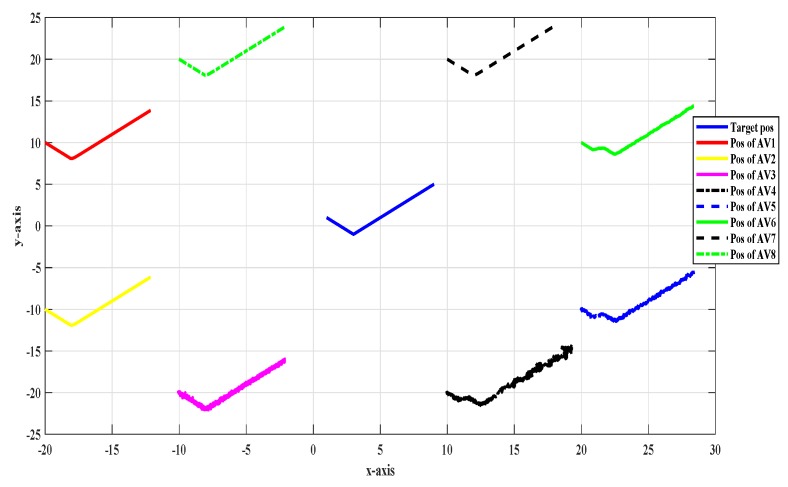
Top of view of the target tracking scenario with CN and LOC faults simultaneously, in the presence of noise and the Kalman filter.

The top view of the target and its follower in which the actual target position, the noisy target position computed by UAV-1, and also the estimated target position by employing the Kalman filter on UAV-1 is shown in Figure 5. First of all, the performance of the Kalman filter was checked in the existence of LOC and CN faults simultaneously. The faults were introduced at an extreme level, that is both the LOC and CN links were broken. It was obvious that the scheme was tested for the worst case scenario in which the follower was neither communicating with the neighbor nor with the target. Here, the CN fault was between UAV-3 and UAV-4, while the LOC fault was present in UAV-5. The proposed work was also tested for different UAVs with different ranges and levels of faults and noise. The top view is shown in Figure 5 with the effect of CN and LOC faults in the presence of noise and the Kalman filter.

**Figure 6:**
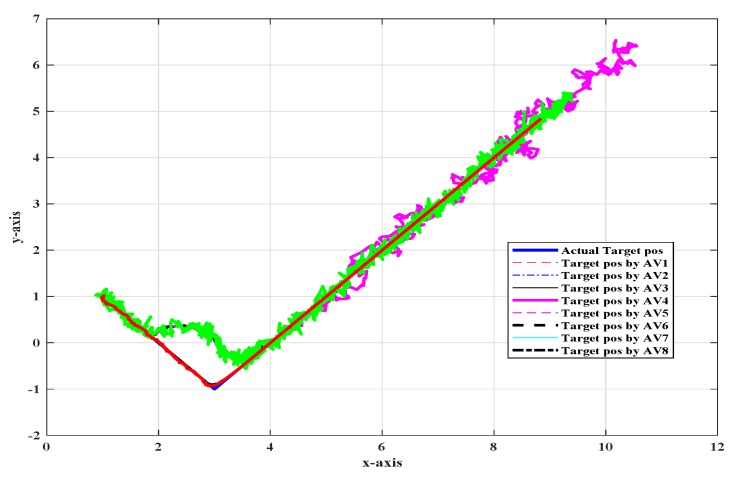
Combine target trajectory with CN and LOC faults, noise, and the Kalman filter.

The target trajectory of UAV-3, UAV-4, and UAV-5 had a single CN and LOC fault simultaneously in the noisy scenario (noise was assumed to be random, zero mean, flat spectral density, and Gaussian), which made it difficult to track the target and predict its position, as shown in Figure 6; to tackle this situation when the noise affected the UAVs, and predict the targets’ position, the Kalman filter was employed to minimize noise and estimate the state vector target position.

The last few plots include the performance analysis of the Kalman filter, which shows the analysis of the Kalman filter in the presence of faults, i.e., single CN and LOC faults simultaneously.

**Figure 7:**
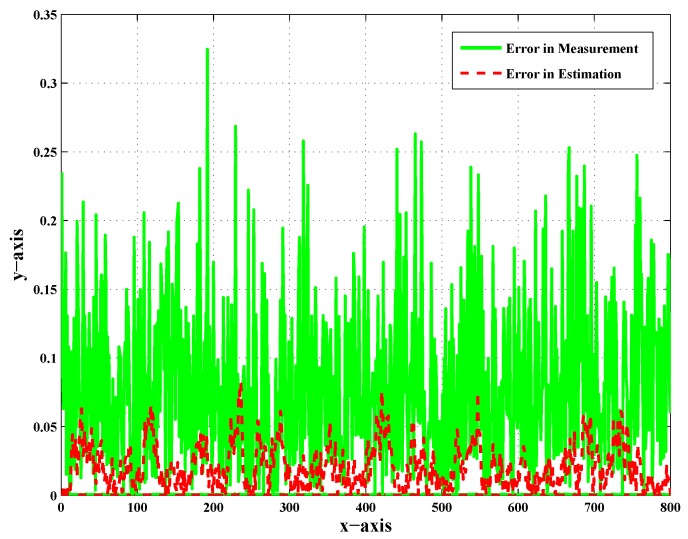
Error in the measurement and error in the estimation of UAV-3 with CN and LOC faults.

**Figure 8:**
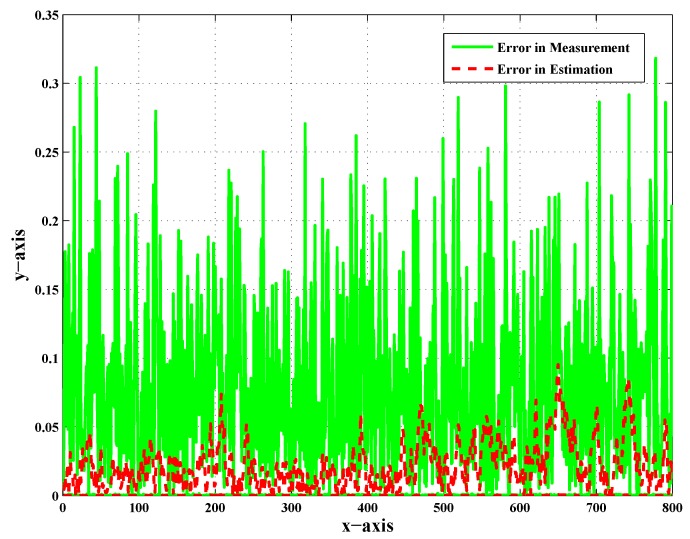
Error in measurement and error in estimation of UAV-4 with CN and LOC faults.

**Figure 9:**
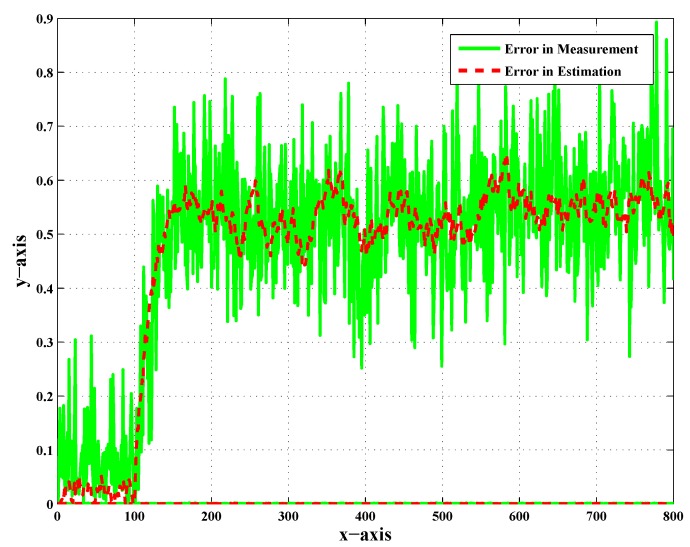
Error in measurement and error in estimation of UAV-5 with CN and LOC faults.

Figure 7, Figure 8 and Figure 9 show the error in measurement and also the error in the estimation of UAV-3, UAV-4, and UAV-5 in particular. These error plots present the deviation of measurement and estimation in meters (m). Figure 9 presents the error in measurement and estimation; it is clear from the figure that the error in measurement showed more deviation from the actual value as compared to the error in the estimation. In addition to that, this was observed as the worst case scenario for faults and noise. A sufficient change in the percentage of error was observed after employing the scheme.

The error in the measurement was large enough, as shown by the green color, before the implementation of the Kalman filter, and because of this, the target was not tracked down by the respective follower UAVs. After employing the Kalman filter in the target tracking scenario, the error was minimized, as shown by the red color. It was concluded that the reduction in the noise effect manifested the performance of the Kalman filter in target tracking. It was also clear that the proposed work effectively helped estimate the target position in the presence of noise and faults. The literature presents effective strategies for dealing with faults in decentralized tracking as discussed in the Introduction. However, in addition to faults, noise was introduced to make it more realistic. In order to deal with such issues, a standard estimation technique was employed. It was an approach employing the Kalman filter in parallel with FDI algorithms to deal with the noise. Acceptable results demonstrated the performance of the proposed scheme.

## 6. Conclusions

Target tracking is the one of the important tasks in the autonomy of vehicles and surveillance. The existing work was to track the target efficiently in the absence of noise. The literature revealed effective tracking in the presence of faults only. This work proposed necessary modifications with the FDI algorithm to deal with the intermittent environment. In addition to this, the Kalman filter was employed to estimate the target position locally at each follower. The scheme was tested for different followers in the presence of faults and noise simultaneously. The proposed work was tested for multiple faults, as well. Simulation with this proposed scheme gave better results, as manifested by the error. This work could be implemented for the tracking of a target by follower UAVs. A target following a linear trajectory could be modeled as an LTI system, as presented in the system model. Estimation in parallel with FDI algorithms were employed, and acceptable results were presented. Future work related to this area is the practical implementation and tracking of nonlinear trajectory movement in the presence of noise. 

## Abbreviations

The following abbreviations are used in this manuscript:

FDI	Fault Detection and Isolation
LTI	Linear Time Invariant
MMSE	Minimum Mean-Squared Error
DPF	Distributed Particle Filtering
CN	Communication with Neighbor
LOC	Line of Communication
UAV	Un-Manned Aerial Vehicle
